# GABA_A_ Receptor β_2_E155 Residue Located at the Agonist-Binding Site Is Involved in the Receptor Gating

**DOI:** 10.3389/fncel.2020.00002

**Published:** 2020-02-11

**Authors:** Magdalena Jatczak-Śliwa, Magdalena Kisiel, Marta Magdalena Czyzewska, Marek Brodzki, Jerzy Władysław Mozrzymas

**Affiliations:** ^1^Laboratory of Neuroscience, Department of Biophysics, Wrocław Medical University, Wrocław, Poland; ^2^Department of Molecular Physiology and Neurobiology, University of Wrocław, Wrocław, Poland

**Keywords:** GABA_A_ receptor, orthosteric binding site, preactivation, structure–function, mutagenesis

## Abstract

GABA_A_ receptors (GABA_A_Rs) play a crucial role in mediating inhibition in the adult brain. In spite of progress in describing (mainly) the static structures of this receptor, the molecular mechanisms underlying its activation remain unclear. It is known that in the α_1_β_2_γ_2L_ receptors, the mutation of the β_2_E155 residue, at the orthosteric binding site, strongly impairs the receptor activation, but the molecular and kinetic mechanisms of this effect remain elusive. Herein, we investigated the impact of the β_2_E155C mutation on binding and gating of the α_1_β_2_γ_2L_ receptor. To this end, we combined the macroscopic and single-channel analysis, the use of different agonists [GABA and muscimol (MSC)] and flurazepam (FLU) as a modulator. As expected, the β_2_E155C mutation caused a vast right shift of the dose–response (for GABA and MSC) and, additionally, dramatic changes in the time course of current responses, indicative of alterations in gating. Mutated receptors showed reduced maximum open probability and enhanced receptor spontaneous activity. Model simulations for macroscopic currents revealed that the primary effect of the mutation was the downregulation of the preactivation (flipping) rate. Experiments with MSC and FLU further confirmed a reduction in the preactivation rate. Our single-channel analysis revealed the mutation impact mainly on the second component in the shut times distributions. Based on model simulations, this finding further confirms that this mutation affects mostly the preactivation transition, supporting thus the macroscopic data. Altogether, we provide new evidence that the β_2_E155 residue is involved in both binding and gating (primarily preactivation).

## Introduction

GABA_A_ receptors (GABA_A_Rs) are essential mediators of inhibitory neurotransmission in the adult mammalian brain and play a critical role in maintaining the correct balance of excitatory and inhibitory signaling that govern the proper function of the nervous system. Dysfunction of these channels leads to many neurological and psychiatric disorders such as epilepsy, anxiety, insomnia, schizophrenia, and autistic spectrum disorders (Bowser et al., 2002; Brambilla et al., 2003; Guidotti et al., 2005; Earnheart et al., 2007; Lewis et al., 2008; Chao et al., 2010; Pizzarelli and Cherubini, 2011). Moreover, GABA_A_Rs are a target for many endogenous and exogenous compounds (including clinically relevant specifics) such as Benzodiazepines (BDZs; Hevers and Lüddens, 1998; Mozrzymas et al., 2007; Wójtowicz et al., 2008; Tan et al., 2011), endozepines (Christian et al., 2013), neurosteroids (Bianchi and Macdonald, 2003; Belelli and Lambert, 2005) barbiturates, and several anesthetics (Krasowski and Harrison, 1999; Rudolph and Antkowiak, 2004). Therefore, it is crucial to understand the molecular mechanisms of the receptor functioning.

Functional GABA_A_Rs are heteropentameric channels co-assembling from a repertoire of 20 subunits (Berezhnoy et al., 2007) while the predominant combination in the vertebrate brain consists of two α_1_, two β_2_, and one γ_2_ subunit (Tretter et al., 1997; Farrar et al., 1999; Farrant and Nusser, 2005). In addition, α and β subunits may co-assemble with δ subunits forming receptors important in, e.g., tonic inhibition, neurosteroid modulation, and alcohol dependence (Wohlfarth et al., 2002; Wallner et al., 2003; Farrant and Nusser, 2005; Belelli et al., 2009; Shu et al., 2012). The neurotransmitter γ-aminobutyric acid (GABA) binds to the receptor at the interface of the α and β subunits (Cromer et al., 2002; Kash et al., 2004; Zhu et al., 2018). The agonist binding triggers the rapid opening of the ion channel permitting a selective flow of anions through the pore. A necessary step in studying the inhibitory neurotransmission is to resolve the molecular mechanisms of GABA_A_R conformational transitions following agonist binding that lead to the channel activation. In spite of a substantial progress in describing mainly the static structures of this receptor (Miller and Aricescu, 2014; Phulera et al., 2018; Zhu et al., 2018), this goal remains far from being achieved. GABA_A_R activation involves movements of its structural elements leading to the transition from a closed to an open conformation of the channel after the agonist binding. Interestingly, neurotransmitter binding sites are very distant from the channel gate (ca. 50 Å) indicating that the mechanism of activation must be very complex, and it may comprise vast parts of the receptor macromolecule (Chakrapani et al., 2004; Miller and Aricescu, 2014). It has been already shown for GABA_A_Rs (Gielen et al., 2012; Szczot et al., 2014; Dixon et al., 2015; Kisiel et al., 2018) and previously also for other receptors of the Cys-loop family (Burzomato et al., 2004; Lape et al., 2008; Mukhtasimova et al., 2009; Jadey and Auerbach, 2012; Corradi and Bouzat, 2014) that, after agonist binding, a key transition prior to channel opening is the preactivation/flipped transition. The preactivated (flipped) state may be thought of as a still closed conformation at which the receptor is poised to undergo an opening transition. Interestingly, at least some key structural determinants (e.g., α_1_F64) of these gating transitions are located very closely to the agonist-binding site (Szczot et al., 2014). It seems thus interesting to pursue the issue of how “strategically” located residues close to the orthosteric binding sites impact the channel gating. Multiple amino acid residues, located on loops A, B, and C of the β subunit and loops D, E, and F of the α subunit, have been identified as important determinants for the GABA-binding site (Wagner et al., 2004; Miller and Smart, 2010; Sander et al., 2011; Miller and Aricescu, 2014). Besides critical involvement of α_1_F64 from loop D in receptor gating (Szczot et al., 2014; Kisiel et al., 2018), there are also some hints (Newell et al., 2004; Mortensen et al., 2014) suggesting that β_2_E155 residue in loop B, may also be involved in channel gating properties, which is clearly manifested by enhancement of the spontaneous activity when mutating it. This residue has been previously shown by the homology modeling of GABA_A_R (Cromer et al., 2002) and ligand docking (Mortensen et al., 2014) to interact with the positively charged moiety of GABA, most probably by anchoring the GABA amino nitrogen end (Zhu et al., 2018). Considering this close interaction of β_2_E155 with GABA, it seems thus particularly interesting to determine how its mutation affects also the gating properties of this receptor when activated by agonists. Moreover, it has been previously shown that mutation in a related subunit β_3_E155G is associated with childhood absence epilepsy (Epi4K and EPGP Investigators, 2013). This fact reveals a particular importance of β_2_E155 residue also in a pathophysiological context.

In the present study, macroscopic and single-channel analyses were applied to address the impact of the β_2_E155 mutation on the α_1_β_2_γ_2L_ channel kinetic properties. We provide evidence that β_2_E155 mutation causes a very substantial binding weakening combined with clear alterations of the gating properties, especially the preactivation transition. The effect of this mutation on the preactivation transition is further supported by the analysis of responses to muscimol (MSC) and by analyzing the modulatory effect of flurazepam (FLU).

## Materials and Methods

### Cell Culture and Expression of Recombinant GABA_A_Rs

For the expression of recombinant GABA_A_Rs, human embryonic kidney cells were used (HEK293 cell line, Sigma-Aldrich), cultured as previously described (Szczot et al., 2014). To transiently transfect the cells, the calcium phosphate precipitation method (Chen and Okayama, 1987) was used. When a stronger expression was needed, the FuGENE HD (Promega) reagent was applied. Rat GABA_A_Rs subunits (α_1_, β_2_, γ_2L_) and mutated β_2_E155C subunit in pUNIV vector were given by Prof. Cynthia Czajkowski from the University of Wisconsin-Madison. The cDNA-encoding human CD4 gene was cloned into the pCMV vector. Expression of a pure fraction of α_1_β_2_E155Cγ_2_ mutants is problematic because of a possibility to express either α_1_β_2_- or α_1_γ_2_-type receptors (Tretter et al., 1997; Brodzki et al., 2016). Typically, to reduce the expression of α_1_β_2_ receptors, the γ_2_ subunit is overexpressed (Boileau et al., 2005; Jatczak-Śliwa et al., 2018; Kisiel et al., 2018, 2019) and therefore, plasmids encoding α_1_, β_2_, and γ_2L_ subunits were added to a transfection solution at a 1:1:3 ratio. However, in the case of the α_1_β_2_E155Cγ_2_ mutants, we observed a marked transfection-to-transfection variability in the time course and amplitude of recorded currents, which could be essentially avoided by the additional overexpression of the β_2_ subunit. Thus, plasmids for α_1_, β_2_E155C, and γ_2L_ subunits were supplied at a ratio of 1:3:3. In the case of mutants, the excess of β_2_E155C was needed to minimize the expression of the functional α_1_γ_2L_ receptors. However, this contamination was relatively easy to be identified. First, the currents mediated by the α_1_γ_2_ receptors, bearing a similar kinetic phenotype to α_1_β_2_γ_2_ wild type (WT) receptors, were characterized by a rapid current onset, whereas currents mediated by the mutants were much slower, and their amplitude was increasing when raising the GABA concentration above 10 mM, which assures saturation for both α_1_β_2_γ_2_ and α_1_γ_2_ receptors (Brodzki et al., 2016). To detect transfected cells, plasmid-encoding CD4 was also added (at the same proportion as plasmids for the α_1_ subunits), and magnetic beads covered with anti-CD4 antibodies (Dynabeads CD4, Thermo Fisher Scientific) were added to the cells prior to recordings. In all transfections, the total amount of DNA was 3 μg. Electrophysiological recordings were performed 24–48 h after transfection.

### Electrophysiological Macroscopic Recordings and Macroscopic Data Analysis

Macroscopic currents were recorded in the whole-cell (lifted cell) configuration of the patch–clamp technique at a holding potential of −40 mV using the Axopatch 200B amplifier (Molecular Devices, Sunnyvale, CA, USA). Because of a marked reduction in the maximum open probability caused by β_2_E155C mutation, we were not able to obtain sufficiently large current responses in the excised patch configuration, which would assure a higher resolution than the whole-cell mode. Signals were low-pass filtered at 10 kHz and sampled at 100 kHz using the Digidata 1440A acquisition card (Molecular Devices, Sunnyvale, CA, USA). For acquisition and analysis the pClamp 10.7 software was used. Patch pipettes were pulled from the borosilicate glass with filament (OD: 1.5 mm, ID: 1.0 mm, Fil: 0.15 mm; Hilgenberg, Malsfeld, Germany), which had resistance of 2.5–5 MΩ, when filled with the internal solution. The intrapipette solution contained (in mM) 137 KCl, 1 CaCl_2_, 2 MgCl_2_, 10 HEPES, 11 EGTA, 2 ATP-Mg, and 10 K-gluconate with pH adjusted to 7.2 with KOH. The external saline consisted of (in mM) 137 NaCl, 20 glucose, 10 HEPES, 5 KCl, 2 CaCl_2_, 1 MgCl_2_, and pH was set to 7.2 with NaOH. For agonist concentration higher than 10 mM, it was necessary to reduce NaCl/KCl concentration to 87 mM to maintain the osmolarity at a constant level. In this case, the external solution was supplemented with glucose and the internal solution with 50 mM K-gluconate (Wagner et al., 2004; Szczot et al., 2014; Kisiel et al., 2019). All experiments were performed at room temperature (20–23°C). All chemicals were bought from Sigma-Aldrich (St. Louis, MO, USA).

For rapid agonist application, the ultrafast perfusion system based on piezoelectric-driven (Physics Instrumente) theta-glass pipettes (Hilgenberg) was used as described in detail by Jonas (1995) and also in, e.g., Mozrzymas et al. (1999), Barberis et al. (2000) and Szczot et al. (2014). Solutions were supplied to the two channels of the theta-glass capillary using an SP220IZ syringe pump (World Precision Instruments, Inc., Sarosta, FL, USA). The open-tip junctional potential onset (10–90% rise time) was in the range of 150–350 μs. Currents mediated by mutated receptors were small, and the highest GABA concentration used (300 mM) was not saturating, corresponding to EC_65_ (determined from Hill equation). Higher GABA concentrations could not be used because of excessive osmolarity imbalance and instability of recordings. Recordings in which the amplitude exceeded 2 nA, the current rundown was larger than 25% or access resistance was larger than 10 MΩ, were excluded from the analysis.

For more elaborate protocols (requiring a larger number of channels supplying different solutions) and when a rapid agonist application was not necessary, a multibarrel rapid solution system RSC-200 (Bio-Logic Science Instruments, Seyssinet-Pariset, France) was used (with exchange time approximately 20–30 ms). This technique was limited to slow signals, specifically to assess the extent of spontaneous activity of mutated receptors. In these experiments, the protocols required several applications of different solutions [3 μM of flurazepam (FLU) and 100 μM of picrotoxin (PTX), open channel blocker] and were performed on adherent cells that showed the highest stability (for more details, see Jatczak-Śliwa et al., 2018).

To determine the differences in dose–response relationships for different receptor types, the measurements of currents evoked by a wide range of agonist [GABA or muscimol (MSC)] concentrations were performed. These data were fitted with the Hill equation: *EC* = 1/(1 + (*EC*_50_/[agonist])^*nH*^), where EC_50_ is the half-maximal concentration, and n_H_ is the Hill coefficient, using the SigmaPlot 11.0 software (Systat Software, San Jose, CA, USA).

The current onset was assessed as 10–90% rise time. For currents mediated by mutated receptors (smaller amplitude and therefore more noisy trace), the onset kinetics was fitted with an exponential function: *y*(*t*) = *A* · (1 *e*^−*t*/*τ*^). The mean rise time 10–90% was calculated then as *t*_rise_ = *τ* · *ln*9.

To study the macroscopic desensitization of currents mediated by mutated receptors, which was characterized by slow kinetics making exponential fitting infeasible, we calculated the FR parameter (fraction remaining) as described previously (Szczot et al., 2014; Jatczak-Śliwa et al., 2018). Briefly, the extent of desensitization was quantified as the relative current remaining 10 ms after the peak (FR10) and after 500 ms of agonist application (FR500).

The kinetics of deactivation was analyzed for currents elicited by two experimental protocols: after a long (500 ms) or short (sufficient to reach the peak; 1–70 ms depending on the receptor type) pulse of agonist. The deactivation of currents for various agonists was fitted by either using a single exponent: *y*(*t*) = *A* · *e*^(−*t*/*τ*)^, where A is the amplitude and τ is the time constant, or with a sum of two exponential functions: *y*(*t*) = *A*_slow_ · *e*^*t*/*τ*_slow_^ + *A*_fast_ · *e*^*t*/*τ*_fast_^, where A_slow_ and A_fast_ are the normalized amplitudes (*A*_slow_ + *A*_fast_ = 1) of slow and fast components, respectively, τ_slow_ and τ_fast_ are the time constants. The mean time constant was determined using the equation: *τ*_mean_ = *A*_slow_ · *τ*_slow_ + *A*_fast_ · *τ*_fast_.

Statistical analysis was performed in Excel 2010 (Microsoft, Redmond, WA, USA) and SigmaPlot 11.0 (Systat Software, San Jose, CA, USA).

### Model Simulations for Macroscopic Currents

For macroscopic currents, model simulations were carried out using ChanneLab2 (Synaptosoft) software. The structure of our model was taken from our previous study (the so-called “flipped” Jones–Westbrook’s model, fJWM; Szczot et al., 2014), with one open and one desensitized fully bound states connected with a fully bound flipped state (Figure 5A). For analysis of ligand-evoked responses, the singly bound states are omitted because of their low probability of occurrence (see “Results” and “Discussion” section). In our experiments, we were not able to achieve the saturation for mutated receptors. The potencies of the agonists used were calculated using extrapolated dose–response relationships, and comparisons were made between currents elicited by agonists with similar potencies. Clearly, to reproduce the time course of such nonsaturating responses, the binding step was taken into consideration. Based on our single-channel modeling where we have made an estimation at 30 μM GABA (EC_32_), singly bound receptors are expected to contribute to approximately only 13% of the events characterized by short open time durations. Thus, singly bound events were considered negligible and omitted in the macroscopic model simulations for 300 mM GABA (EC_65_), 300 mM GABA + 3 μM FLU and 30 mM MSC (EC_68_) for mutated receptors. In the present study, to reproduce our experimental data for wild-type receptor, we used the rate constants from the fJW model (Szczot et al., 2014). To reproduce our results for the β_2_E155C mutant, the rate constants were selected to best reproduce the dose–responses and, at the same time, kinetic features of the recorded current (amplitudes, onset kinetics, fading, and deactivation) for GABA, MSC, and GABA + FLU application for mutated receptors.

**Figure 1:**
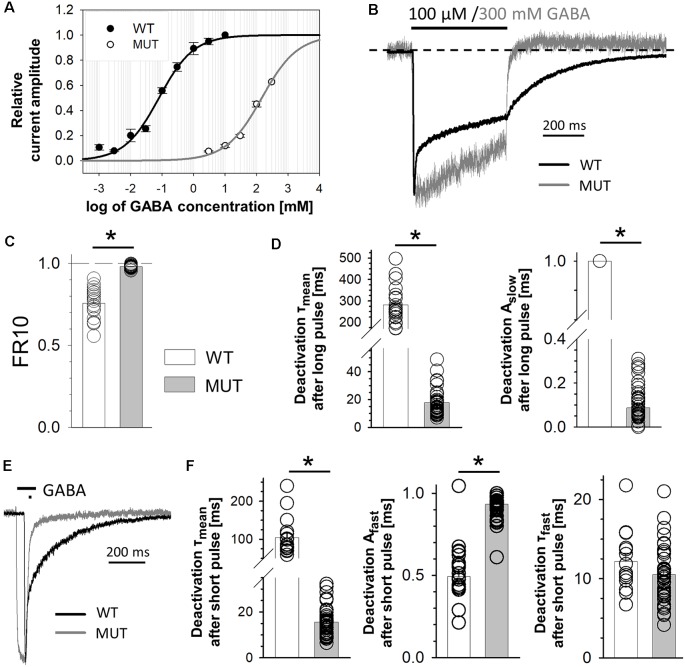
The impact of β_2_E155C mutation on GABA-evoked macroscopic currents. **(A)** GABA dose–response relationships with fitted Hill’s curves for α_1_β_2_γ_2_ [wild type (WT), EC_50_: 79.2 μM, n_H_: 0.79] and α_1_β_2_E155Cγ_2_ [mutated GABA_A_ receptors (GABA_A_Rs; MUT), EC_50_: 146 mM, n_H_: 0.76] GABA_A_Rs. Note that this mutation strongly right shifted the dose–response dependence. **(B–D)** The impact of mutation on the kinetics of macroscopic currents evoked by a prolonged (500 ms) application of GABA. **(B)** Typical traces of normalized current responses mediated by WT (black) and MUT (gray) receptors to pulses of 100 μM and 300 mM GABA, respectively. Baseline level is marked with a dashed line to make a spontaneous activity-related current overshoot (after agonist removal, see Jatczak-Śliwa et al., 2018) clearly visible. Note a strong effect of mutation on macroscopic desensitization. **(C)** Statistics for the fraction of current remaining after 10 ms from the peak. Note that mutation abolishes the fast component of macroscopic desensitization. **(D)** Statistics of deactivation kinetics after long (500 ms) GABA application—mean time constant (τ_mean_, left) and percentage of the slow component (A_slow_, right). **(E,F)** The impact of mutation on deactivation kinetics after short (few ms) GABA application. **(E)** Typical traces of macroscopic currents elicited by short pulses of GABA mediated by WT (black) and MUT (gray) receptors. **(F)** Statistics of deactivation kinetics after short pulses of GABA—mean time constant (τ_mean_, left), percentage of the fast component (A_fast_, middle) and its time constant (τ_fast_, right). Note that mutation accelerates deactivation kinetics after long pulse mainly by decreasing the percentage of slow deactivation phase while in the case of deactivation after short agonist application also by decreasing the time constant of the fast component. Asterisks indicate statistically significant differences.

**Figure 2:**
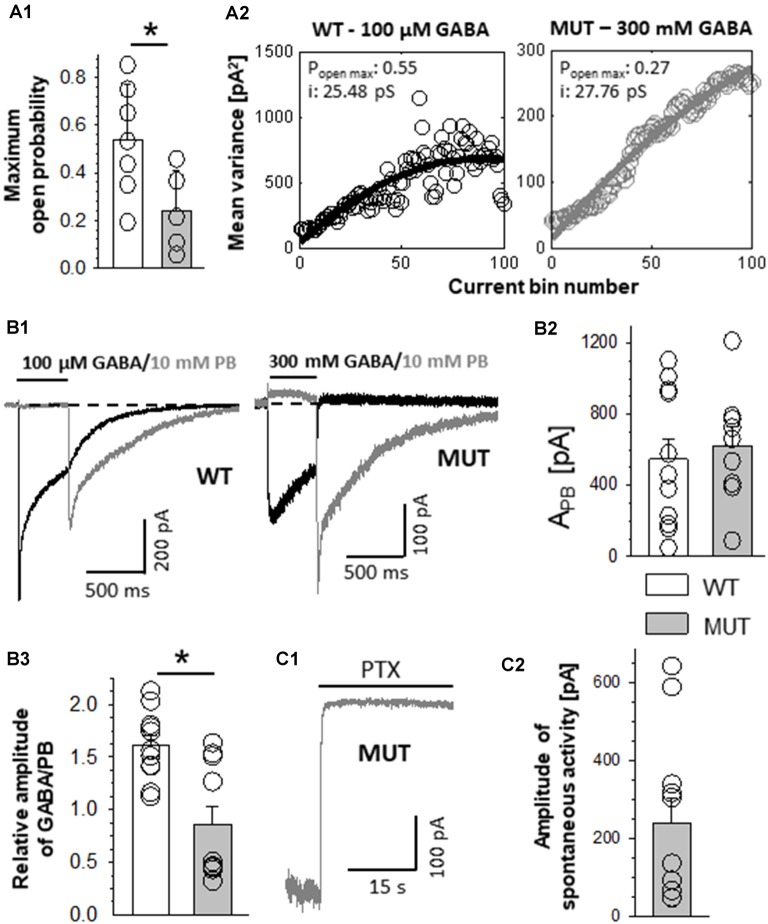
The impact of β_2_E155C mutation on the maximum open probability and the level of spontaneous activity. **(A1)** Statistics of the maximum open probability determined using nonstationary variance analysis (NSVA). **(A2)** Plots that represent the variance of the current at each time point. **(B)** Results of experiments in which high (GABA) or 10 mM PB was used. **(B1)** Left, typical traces of current responses evoked by 100 μM GABA (black line) and rebound current observed upon removal of 10 mM PB (gray line) mediated by wild-type (WT) receptors. **(B1)** Right, currents mediated by the mutants (MUT) and evoked by 300 mM GABA and tail currents following wash-out of 10 mM PB. Insets above traces indicate agonist applications. Baseline level is drawn with a dashed line. Note that in the case of mutation, a larger block appearing during PB application is seen, which indicates a larger extent of spontaneous current mediated by the MUT than in the case of WT receptors. **(B2)** Statistics of the absolute amplitudes of tail currents observed after PB removal for compared receptors. **(B3)** Statistics of the ratios between GABA-evoked currents and tail currents after PB removal for WT and MUT receptors. GABA and MSC-evoked currents were measured from the same cell. Note that whereas the amplitudes of tail currents measured after removal of PB were similar in WT and in MUT **(B1,B2)**, the current ratio (GABA/PB, **B3**) was markedly larger in the case of WT receptors. Asterisks indicate a statistically significant difference. **(C1)** Typical trace of spontaneous current mediated by mutated GABA_A_Rs (MUT) blocked by 100 μM of picrotoxin (PTX). **(C2)** Statistics for the amplitude of spontaneous activity for MUT. Note that β_2_E155C mutation results in a particularly large spontaneous activity of GABA_A_Rs (spontaneous activity determined in exactly the same experimental conditions for WT receptors was 15.5 ± 2.8 pA, *n* = 13; Jatczak-Śliwa et al., 2018).

**Figure 3:**
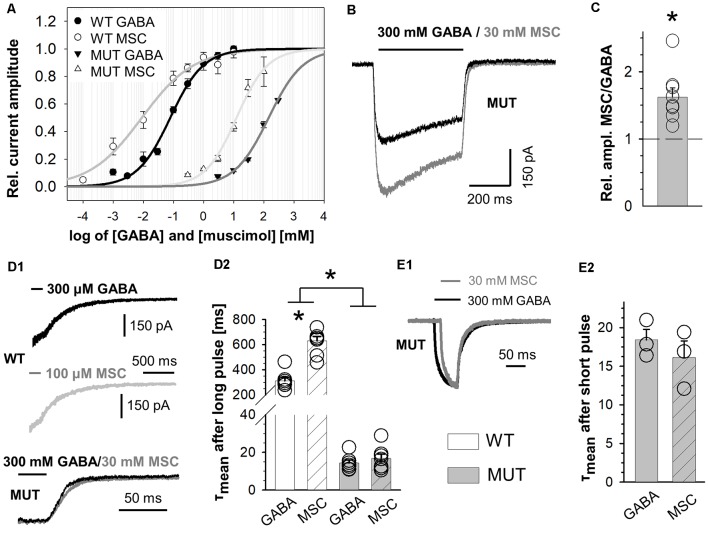
The impact of β_2_E155C mutation on kinetics of muscimol (MSC)-evoked macroscopic currents. **(A)** Dose–response relationships with fitted Hill’s equation for α_1_β_2_γ_2_ (WT-MSC: EC_50_: 9.0 μM, n_H_: 0.50) and α_1_β_2_E155Cγ_2_ (MUT-MSC: EC_50_: 12.5 mM, n_H_: 0.84) GABA_A_Rs (GABA—black symbols and MSC—white symbols). Note that the mutation strongly right shifted the dose–responses for GABA, while for MSC, the shift was qualitatively similar, considering that the dose–response for MSC shows a leftward shift with respect to that for GABA both for WT and for mutated receptors. **(B)** Typical traces of current responses mediated by MUT receptors evoked by long pulses of 300 mM GABA (black) and 100 mM MSC (gray) measured from the same cell. **(C)** Statistics of relative amplitudes (MSC/GABA) for mutated receptors. Each point on the plot represents recordings made from the same cell. Note that in spite of a similar potency of MSC and GABA, the former agonist evokes currents with absolute amplitude larger by nearly 50% than in the case of GABA. **(D,E)** The impact of β_2_E155C mutation on deactivation kinetics after prolonged **(D)** and short **(E)** application of agonists (for WT: 300 μM GABA and 100 μM MSC). **(D1,E1)** Typical traces showing deactivation phases of current responses to 500-ms agonist application **(D1)** or to short (few ms) pulses of these ligands **(E1)**. Currents evoked by GABA are drawn with a black line, whereas those for MSC with a gray one.** (D2,E2)** Statistics for the mean deactivation time constant (τ_mean_) after long **(D2)** and short **(E2)** pulses of MSC (crosshatch) or GABA (unfilled). In the statistics graphs **(D2,E2)**, bars referring to WT receptors are white, and those describing mutants are gray. Note that β_2_E155C mutation abolishes differences between deactivation kinetics for currents evoked by GABA and MSC. Asterisks indicate a statistically significant difference.

**Figure 4:**
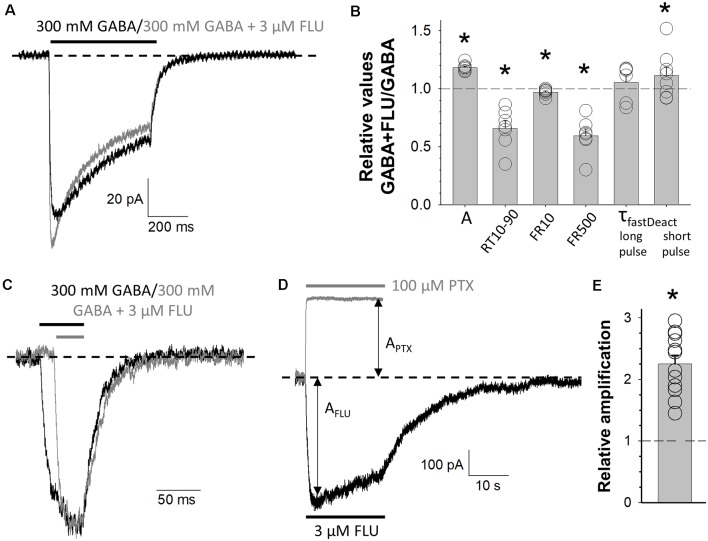
Modulation of mutated receptors (MUT) by 3 μM of flurazepam (FLU). Typical traces of MUT-mediated current responses evoked by 300 mM GABA (black) and 300 mM GABA + 3 μM FLU (gray) for long **(A)** and short **(C)** application where the baseline level is marked with a dashed line. **(B)** Statistics for relative values (300 mM GABA + 3 μM FLU vs. 300 mM GABA): A—amplitude of currents, RT 10–90—current onset, FR10—fraction of current remaining after 10 ms from peak, FR500—fraction of current remaining after 500 ms from the beginning of agonist application, τ_fast_—time constant of the fast component of deactivation kinetics for long or short agonist application. **(D)** Typical traces for MUT-mediated current responses for 3 μM FLU alone (black) or 100 μM picrotoxin (PTX, gray) application. Respective amplitudes are marked with arrows and the baseline level with a dashed line. **(E)** Relative enhancement of spontaneous activity by FLU determined as (A_FLU_ + A_PTX_)/A_PTX_, where A_FLU_ is the amplitude of current responses to 3 μM of FLU application, and A_PTX_ is the amplitude of spontaneous activity calculated as the extent of block by 100 μM PTX (see “Materials and Methods” section). Asterisks indicate a statistically significant difference.

**Figure 5:**
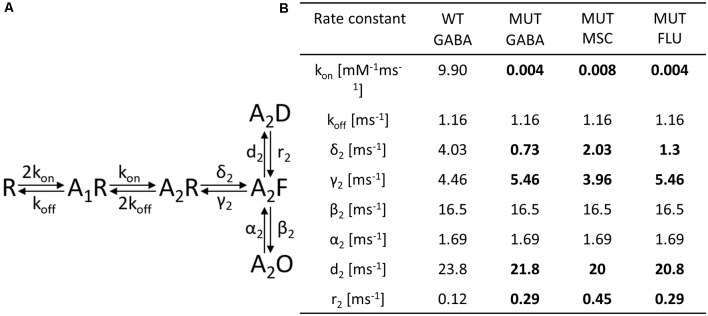
Kinetic model simulations for macroscopic currents analysis. **(A)** Macroscopic kinetic model framework for GABA_A_R used for our simulations (Szczot et al., 2014). **(B)** Table presenting the rate constant values, for GABA-evoked responses for wild-type (WT) receptor and for currents mediated by mutated (MUT) receptors (GABA, GABA + FLU, and MSC). Values in bold font indicate rate constants for mutants for which trend analysis indicated changes with respect to WT-GABA.

### Nonstationary Variance Analysis

Considering that macroscopic recordings (rapid agonist application) are performed in dynamic conditions, whereas single-channel experiments are made in steady-state conditions, it is of interest to be able to compare the kinetic features of the receptors in these two situations (Kisiel et al., 2019). To assess the maximum open probability (P_openMax_) in the dynamic conditions, we have used NSVA (Sigworth, 1980) for currents elicited by rapid agonist application. NSVA was performed as described previously (Szczot et al., 2014). Briefly, ≥10 consecutive responses to short application of high agonist concentration were recorded from the same cell. For the NSVA, the custom MatLab program (Mathworks) was used. Current amplitude (A) and noise variance (σ^2^) were calculated for each time point from peak to baseline (De Koninck and Mody, 1994). The values of the current amplitude were divided into 100 equal bins, and the corresponding variances were averaged. Variance was plotted vs. the mean current and fitted with the equation: *σ*^2^ = *iA* − *A*^2^ · *N*^−1^ + *c*, where *i* is the single-channel current, *N* is the number of channels, and *c* is the baseline noise (Ghavanini et al., 2006). The maximum open probability (P_openMax_) was determined using the equation: *P*_openMax_ = *A*_Peak_ · (*i* · *N*)^−1^.

### Single-Channel Recordings

Single-channel currents were recorded in the cell-attached patch-clamp configuration at a 100-mV holding potential. Signals were amplified by an Axopatch 200B amplifier (Molecular Devices, Sunnyvale, CA, USA) and digitized by Digidata 1550B acquisition system (Molecular Devices, Sunnyvale, CA, USA). Signals (free-run sweeps lasting for a few minutes) were low-pass filtered (10 kHz) and sampled at 100 kHz. For WT receptors, 30 μM GABA was used, for MUT: 100 mM GABA and 8 mM MSC. In the case of agonist concentrations below 10 mM, external and internal solutions for single-channel recordings consisted of (in mM) 102.7 NaCl, 20 Na-gluconate, 2 CaCl_2_, 2 KCl, 1.2 MgCl_2_, 10 HEPES, 20 TEA-Cl, 14 D(+)-glucose, 15 Sucrose, pH adjusted to 7.4 by 2 M NaOH, with agonist applied to the intrapipette solution. For 100 mM GABA applications, low-chloride solutions were used with (in mM) 70 NaCl, 10 Na-gluconate, 2 CaCl_2_, 2KCl, 1.2 MgCl_2_, 10 HEPES, 20 TEA-Cl, and D(+)-glucose (to obtain similar osmolarity as solutions with lower agonists concentrations). All experiments were performed using pipette electrodes pulled from borosilicate glass capillaries (OD: 1.5 mm, ID: 0.87 mm; Hilgenberg, Malsfeld, Germany). To obtain a minimal level of signal noise, pipettes were coated with Sylgard 184 (Dow Corning, Auburn, MI, USA) and fire polished before filling with internal solution. Pipettes used in recordings had a resistance of 6–10 MΩ.

### Analysis of Single-Channel Currents

Single-channel kinetic analysis was performed using SCAN and EKDIST software (DCProgs), which was given to our group by David Colquhoun (UCL, London). As described in detail in our recent study (Kisiel et al., 2018, 2019), single-channel traces (stored in the form of *.abf—Axon Binary File) selected for analysis contained ~10,000 events. Filtering of the trace was performed in two stages. First, the recorded signal was filtered with an analog filter (mounted on the amplifier Axopatch 200B) with cutoff frequency of 10 kHz. Next a digital filtering (eight-pole Bessel filter by pClamp software) was applied to achieve the signal-to-noise ratio of at least 15:1 (unitary current divided by the SD of the baseline noise). The cutoff frequency of the digital filter depended on the trace quality and was typically in the range 1.67–2.5 kHz. The final cut off frequency (f_c_) was calculated as 1/f_c_ = 1/f_a_ + 1/f_d_, where f_a_ is the analog filter frequency (10 kHz), and f_d_ is the digital filter frequency. Finally, the sampling frequency (f_s_) was reduced to f_s_ = 10 f_c_. Typically, in our recordings, a resolution of 40 ms could be achieved with a 2.5 kHz filter (f_a_) and 25 kHz sampling rate (f_s_). Recordings with multilevel openings were excluded from the analysis. The idealization (open/closed) of single-channel activity was performed using the SCAN software, and the information on the open and closed states was stored in the *.scn files. The strength of approach used in the SCAN software (see for details http://www.onemol.org.uk/dcmanuals.pdf by Dr. David Colquhoun) is that it performs a continuous time course fitting of a single-channel trace taking into consideration the impact of filtering (whose parameters are specified prior to analysis). This approach greatly increases the capacity to detect short and small events, which, in the standard 50% threshold algorithm, would be undetected. In addition, each detected event is manually inspected by an investigator so the artifacts can be separated from genuine single-channel events. Afterward, these files were processed with EKDIST to create dwell-time distributions for open and shut events. Each of these distributions was then fitted with the sum of exponentials, and the respective time constants (τ) and percentages (P) were determined. To assess the differences in the single-channel conductances between various experimental groups, the mean current amplitudes recorded at a holding potential of 100 mV were compared. This comparison has been made under assumption that the membrane potential of the HEK293 cells does not show group-to-group variability.

### Model Simulations for the Single-Channel Activity

The kinetic simulations of single-channel recordings were performed using the HJCFIT software (DCprogs, maximum likelihood method). The model framework was based on that proposed in our recent study—with two open and two desensitized fully bound states—model 1 from Kisiel et al. (2018) and in the present article (Figure 7A). As was mentioned above, in the case of single-channel recordings, where excessive osmolarity could affect stability of cell-attached patches, we had to use even lower agonist concentrations (WT—30 μM GABA, EC_32_; MUT—100 mM GABA, EC_43_, and 8 mM MSC, EC_41_). In our previous study (Kisiel et al., 2018), we assessed the percentages of doubly bound, singly bound, and spontaneous activity GABA_A_R activity evoked by different GABA concentrations. However, as already discussed above, in the case of WT receptors, even at 30 μM GABA, contribution from singly bound openings was negligible, and we focused our analysis on doubly bound single-channel events.

**Figure 6:**
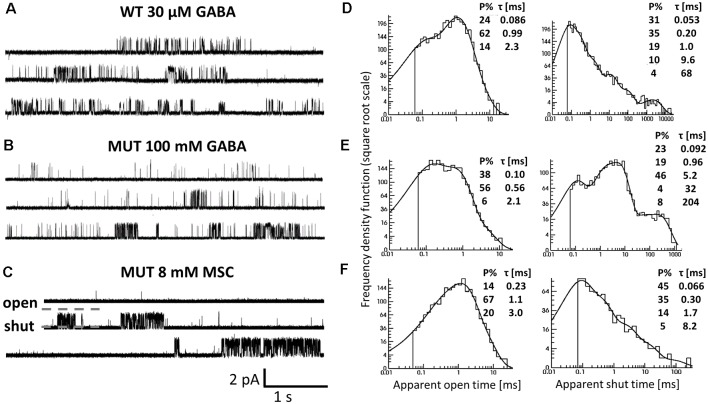
The impact of β_2_E155C mutation on the single-channel activity of α_1_β_2_γ_2_ receptors. **(A–C)** Typical traces of single-channel currents mediated by WT receptors evoked by 30 μM GABA **(A)** and for mutants (MUT) elicited by 100 mM GABA **(B)** and 8 mM MSC **(C)**. **(D–F)** Typical dwell-time distributions (left: openings, right: closures) for single-channel recordings presented in **(A–C)**. Insets in each distribution presents time constants (τ) and percentages (P*%* > 1) of specified components.

**Figure 7:**
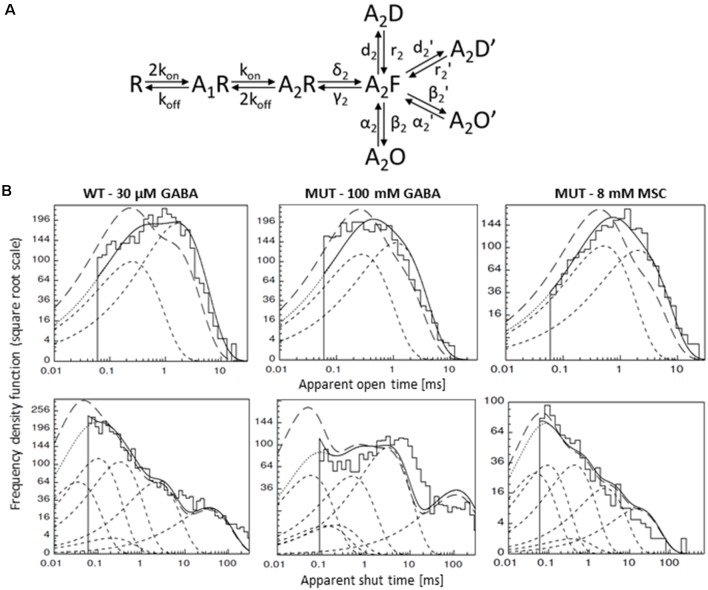
Kinetic model simulations for the single-channel activity. **(A)** Single-channel kinetic model scheme describing GABA_A_R states. Model scheme is from Kisiel et al. (2018). **(B)** Typical dwell-time distributions (top: openings, bottom: closures) for single-channel recordings (left—WT GABA, middle—MUT GABA, right—MUT MSC) with curves showing distributions simulated with rate constants presented in Table 2: with experimental resolution (solid lines), after correction for missed events (long-dashed lines) and for single transitions (dashed lines).

## Results

### Mutation of β_2_E155 Residue Shifts the Dose–Response Curve and Affects Receptor Macroscopic Kinetics

In the first step, we checked how the cysteine mutation of the β_2_E155 residue affected the receptor responsiveness to GABA. As expected, this mutation caused a strong rightward shift in the dose–response relationship (Figure 1A). The loss of GABA sensitivity was so robust that saturation was not reached even at 300 mM GABA (Figure 1A), which was the largest concentration we could use in these experiments (see “Materials and Methods” section). Nevertheless, we have extrapolated this dose–response by fitting the Hill’s equation, which yielded EC_50_ = 146 mM, i.e., roughly 2,000-fold lower potency of GABA compared to wild-type receptors (EC_50_ = 79.2 μM, which is in qualitative agreement with Brodzki et al. (2016; Figure 1A). This effect of mutation on the potency is in accordance with previous findings (Newell et al., 2004; Mortensen et al., 2014). However, it needs to be considered that the dose–response shift can be caused not only by changes in ligand binding but also in the channel gating properties (Colquhoun, 1998), and these contributions might be not easy to be extracted from the dose–response alone. In order to characterize the impact of the β_2_E155C mutation on binding and gating properties, we have analyzed the time course of currents mediated by mutated receptors and elicited by rapid agonist applications. Gating properties are typically studied under a condition of saturation, but in the case of this mutant, as already mentioned, it could not be achieved as the maximal dose of GABA (300 mM) corresponded to approximately EC_65_. Considering this limitation, we compared the current responses to 300 mM GABA for the mutant with currents evoked by a nearly equipotent GABA concentration (100 μM—EC_54_) for the WT receptors (Figure 1B). This small difference in GABA potencies when activating WT and mutated receptors (EC_65_ vs. EC_54_) was taken into account in model fitting by considering the binding steps for these receptors and by choosing GABA concentrations reproducing these potencies. As shown in Figure 1B, mutation of β_2_E155 residue practically eliminates the rapid component of the macroscopic desensitization with remaining slow component. Considering this difference, which precludes exponential fitting to the fading phase of currents mediated by mutants, instead of time constants, the FR10 parameter was used (fraction of current remaining 10 ms after the peak, see “Materials and Methods” section) to characterize macroscopic desensitization, which in the case of mutated receptors was close to 1 (0.98 ± 0.002, *n* = 41), and for WT receptors, it was significantly smaller (0.76 ± 0.02, *n* = 20; *p* < 0.001; Figure 1C).

The current responses mediated by α_1_β_2_E155Cγ_2L_ receptors had a particularly fast deactivation kinetics measured after a long (500 ms) or short (a few ms) GABA application (τ_mean_ = 17.8 ± 1.3 ms, *n* = 45 for long pulse and τ_mean_ = 15.5 ± 1.1 ms, *n* = 36 for short pulse) when compared to the WT receptors (for long application, τ_mean_ = 281.5 ± 19.4 ms, *n* = 20; vs. MUT *p* < 0.001; for short application, τ_mean_ = 104.8 ± 11.8 ms, *n* = 17; vs. MUT *p* < 0.001; Figures 1D–F). In the case of long agonist application, we could observe only one—slow component of deactivation kinetics for WT receptors (specified above as τ_mean_), whereas for mutants, also a fast component was present, which was predominant, and the contribution of the slow one was small (τ_slow_ = 123.8 ± 18.4 ms, *n* = 33; A_slow_ = 0.09 ± 0.01; both vs. WT *p* < 0.001; Figure 1D). Deactivation after short application of GABA (Figure 1E) in both receptor types was characterized by two components with significantly different slow time constants and similar fast ones (WT: τ_slow_ = 190.5 ± 12.2 ms, *n* = 17; τ_fast_ = 12.2 ± 1.0 ms, *n* = 14; MUT: τ_slow_ = 115.8 ± 14.9 ms, *n* = 28, vs. WT *p* = 0.001; τ_fast_ = 10.5 ± 0.6 ms, *n* = 36, vs. WT *p* = 0.17). In addition, their contributions are significantly different (WT: A_slow_ = 0.51 ± 0.03, *n* = 17; A_fast_ = 0.49 ± 0.03, *n* = 17; MUT: A_slow_ = 0.15 ± 0.05, *n* = 29; A_fast_ = 0.93 ± 0.01, *n* = 36; both *p* < 0.001; Figures 1E,F). Altogether, these macroscopic data reveal robust differences in the time courses of currents mediated by the WT receptors and by mutants strongly indicating alterations in the gating mechanisms.

### Mutation of β_2_E155 Residue Reduces the Maximum Open Probability Without Affecting the Single-Channel Conductance

While completing our data based on macroscopic recordings in the whole-cell mode, we observed that the currents mediated by mutants were considerably smaller than those mediated by the WT receptors. This might be due to either a lower expression level, lowered open channel probability, or a decreased single-channel conductance. To assess the maximum open channel probability (P_openMax_) in dynamic conditions of rapid agonist applications, we used NSVA. It is worth emphasizing that single-channel recordings (see subsequent sections) reveal the open probability in the stationary, not dynamic conditions. Our NSVA indicate a significant decrease in P_openMax_ caused by E155C mutation compared to WT (WT: 0.54 ± 0.09, *n* = 7; MUT: 0.24 ± 0.08, *n* = 5, *p* = 0.04; Figure 2A1). NSVA provides also an estimate of the single-channel conductance, which was unaffected by the mutation (WT: 30.7 ± 4.0 pS, *n* = 7; MUT: 34.5 ± 2.3 pS, *n* = 5, *p* = 0.48; Figure 2A2, which shows typical plots representing the variance of the current at each time point). The lack of difference between single-channel conductances of WT receptors and mutants is further supported by our single-channel recordings (see subsequent sections).

In order to provide an estimate of GABA_A_R expression level, we examined the activation of these receptors by pentobarbital (PB). Barbiturates are known to bind to a site that is much closer to the channel gate than the orthosteric binding site, and it is expected that GABA_A_R activation by PB is not markedly affected by the considered mutation (at GABA binding site). It needs to be additionally considered that although high PB concentrations (millimolar range) activate GABA_A_Rs (Steinbach and Akk, 2001), a very efficient open channel block takes place, too (Gingrich et al., 2009). Thus, upon PB application, no current is seen but the extent of GABA_A_R activation is revealed by the so-called rebound currents, which appear upon PB removal (Figure 2B). For each cell expressing WT or mutant receptors, we recorded both GABA-evoked responses (100 μM for WT and 300 mM for MUT) and currents elicited by 10 mM PB (Figure 2B1). Interestingly, amplitudes of PB-evoked currents did not show any significant differences between cells expressing WT receptors and mutants (WT: A_PB_ = −568 ± 127 pA, *n* = 11; MUT: A_PB_ = −645 ± 98 pA, *n* = 10, *p* = 0.64; Figure 2B2). This finding indicates that the expression level of WT receptors and mutants is comparable.

In the case of cells expressing WT receptors, the amplitude of GABA-evoked currents relative to PB rebound is considerably larger than that determined for the mutant receptors (GABA/PB ratio for WT: 1.61 ± 0.10, *n* = 11; MUT: 0.86 ± 0.17, *n* = 10, *p* = 0.001; Figure 2B3). These findings provide additional evidence for P_openMax_ decrease caused by β_2_E155C mutation when activating these receptors with GABA.

### β_2_E155 Mutation Enhances the Receptor Spontaneous Activity

WT α_1_β_2_γ_2_ receptors are characterized with a very low spontaneous activity (Shin et al., 2017; Jatczak-Śliwa et al., 2018; Kisiel et al., 2018). In the present study, we assessed the extent of spontaneous activity using two different compounds: PB and picrotoxin (PTX). The former one was used at a concentration of 10 mM, at which the blocking effect predominates revealing thus the extent of spontaneous activity as an offset of the baseline activity (Figure 2B1, for mutants). Notably, PB-induced current offset was substantial (10.9 ± 3.5% relative to rebound current, *n* = 11) for currents mediated by β_2_E155 mutants, whereas in the case of WT, it could be barely seen (Figure 2B1, for WT) indicating a considerably larger spontaneous activity in the case of mutants. A similar effect was found when using a different open channel blocker—PTX (100 μM). Using this compound, in our recent study, we found that the spontaneous activity of the WT receptors is small (15.5 ± 2.8 pA, *n* = 13; Jatczak-Śliwa et al., 2018), which is in agreement with the above-mentioned negligible spontaneous current when applying PB at a high concentration (Figure 2B1, left). Notably, in the case of β_2_E155 mutants, PTX application resulted in a large current offset (A_PTX_ = 240 ± 66 pA, *n* = 11; Figures 2C1,C2) being ~15-fold larger compared to the WT receptors (see Jatczak-Śliwa et al., 2018). It has to be emphasized that we have not found a significant difference between the estimated number of channels for WT (*N* = 2667 ± 628) and MUT (*N* = 1185 ± 328, vs. WT *p* = 0.09); thus, the higher blocking PTX effect can be ascribed to an increased spontaneous activity. Moreover, such a vast spontaneous activity of mutants is also manifested as a characteristic overshoot, which occurs when recording macroscopic currents upon agonist removal (Figure 1B). Our findings regarding increased spontaneous activity upon β_2_E155 mutation are in qualitative agreement with previous studies by Newell et al. (2004) and Mortensen et al. (2014) for both α_1_β_2_ and α_1_β_2_γ_2S_ receptors.

### Muscimol Acts as a Superagonist on β_2_E155 Mutants

In the case of WT receptors, MSC acted as an agonist with particularly high affinity leaving the gating unchanged with respect to GABA (Jones et al., 1998). However, in the case of the α_1_F64 mutation, this compound acted as a superagonist (Szczot et al., 2014) shedding light on the impact of this mutation on the receptor gating (especially preactivation), and we applied a similar strategy for the β_2_E155 mutant. The dose–response constructed for α_1_β_2_E155Cγ_2L_ receptors activated by MSC was markedly shifted to the left with respect to GABA (Figure 3A). However, the maximum MSC concentration that could be used is 100 mM, and it was still missing the saturation by a relatively minor margin, and the fitting procedure (to extrapolate dose–response) was needed (Figure 3A). In our experiments, we compared the kinetic features of responses elicited by nearly equipotent concentrations of MSC (30 mM—EC_68_) and GABA (300 mM—EC_65_) and compared these results with those for 300 μM GABA (EC_74_) and 100 μM MSC (EC_77_) applied to WT receptors. As expected (Jones et al., 1998), the current amplitudes mediated by the WT receptors and elicited by saturating GABA and MSC had very similar amplitudes. However, for mutated receptors, the amplitude of currents elicited by 30 mM MSC was significantly larger than that for 300 mM GABA (A_MSC/GABA_ = 1.62 ± 0.14, *n* = 8, *p* = 0.008). Notably, this 62% increase exceeds, by far, a tiny difference (3%) in the potencies of these concentrations of GABA and MSC. It needs to be stressed that GABA and MSC concentrations used here were relatively close to saturation (missing saturation by 32% and 35%) clearly indicating that MSC acts here as superagonist-activating mutated receptors with a mechanism characterized by different gating features than in the case of GABA. Moreover, besides this effect on current amplitudes, in the case of mutants, we have not observed any significant difference in the deactivation kinetics of currents evoked by MSC or GABA, while for WT receptors, MSC-elicited currents had deactivation twice as long as in the case of GABA (for long pulse: WT, 300 μM GABA: τ_mean_ = 312.5 ± 27.2 ms, *n* = 7, 100 μM MSC: τ_mean_ = 630.4 ± 34.9 ms, *n* = 8, vs. GABA *p* < 0.001; MUT, 300 mM GABA: τ_mean_ = 24.4 ± 7.9 ms, *n* = 8, 30 mM MSC: τ_mean_ = 22.8 ± 8.5 ms, *n* = 8 vs. GABA *p* = 0.224; for short-pulse MUT: 30 mM MSC: τ_mean_ = 31.1 ± 15.0 ms; 300 mM GABA: τ_mean_ = 34.5 ± 16.1 ms, *n* = 4, *p* = 0.15; Figures 3D,E).

### Effect of Flurazepam Reveals Impact of E155 Mutation on Receptor Gating

BDZs are positive modulators of GABA_A_Rs, and their mechanism of modulation involves agonist binding as well as channel gating transitions (Rüsch and Forman, 2005; Campo-Soria et al., 2006; Mercik et al., 2007; Mozrzymas et al., 2007; Li et al., 2013; Goldschen-Ohm et al., 2014; Dixon et al., 2015; Jatczak-Śliwa et al., 2018). In particular, our recent study indicates that FLU alters the receptor gating mainly by affecting the preactivation and desensitization properties (Jatczak-Śliwa et al., 2018). In order to pursue the mechanism whereby mutation of the β_2_E155 residue affects the channel gating, we investigated the impact of FLU modulation on macroscopic current kinetics of these mutants. To this end, we confronted the kinetics of current responses elicited by GABA (300 mM) in the presence or absence of 3 μM of FLU. As shown in Figures 4A,B, FLU significantly increased current amplitude (A_FLU+GABA_/A_GABA_ = 1.18 ± 0.01, *n* = 7, *p* = 0.034). Moreover, we found a significant acceleration of the rise time (RT10–90_FLU+GABA_/RT10–90_GABA_ = 0.66 ± 0.06, *n* = 7, *p* = 0.016) as well as a reduction in macroscopic desensitization parameters FR10 and FR500 (FR10_FLU+GABA_/FR10_GABA_ = 0.97 ± 0.01, *n* = 7, *p* = 0.032; FR500_FLU+GABA_/FR500_GABA_ = 0.60 ± 0.06, *n* = 7, *p* < 0.001; Figure 4B). Deactivation kinetics was also altered, but significant (although minor) slowdown effect was observed only in the case of short-pulse application (τ_fastFLU+GABA_/τ_fastGABA_ = 1.12 ± 0.07, *n* = 7, *p* = 0.04; Figures 4B,C). In addition, we have shown that FLU enhanced the spontaneous activity of mutated receptors [(A_PTX_ + A_FLU_)/A_PTX_ = 2.23 ± 0.15, *n* = 11, *p* < 0.001] (Figures 4D,E). Interestingly, the extent of FLU-induced increase in the spontaneous activity for the considered mutant was similar to those previously observed for WT and α_1_F64 mutants (see Jatczak-Śliwa et al., 2018).

### Model Simulations for Macroscopic Currents Demonstrate That β_2_E155 Mutation Alters Binding and Preactivation

To further explore the mechanism whereby the β_2_E155 mutation alters the GABA_A_R activation, model simulations were used. For this purpose, we considered the kinetic model previously used by our group (Figure 5A; Szczot et al., 2014). First, we made an attempt to choose appropriate rate constants allowing to replicate experimentally observed time course of responses to 300 mM GABA mediated by mutated receptors. Considering that the highest GABA concentration used (300 mM) was not saturating (approximately EC_65_), we had to assess both binding and gating rate constants. For this purpose, we had to reproduce at the same time the dose–response relationship and the time course of currents mediated by the mutants and evoked by 300 mM GABA. Reproduction of the dose–response required primarily a very large reduction of the binding rate parameter, whereas the time course of currents evoked by 300 mM GABA marked, and significant changes in the gating rate constants were needed (Figure 5B). The major kinetic findings for responses mediated by mutants and evoked by 300 mM GABA were a robust slow down of the macroscopic desensitization and acceleration of deactivation process. In our simulations, these observations could be fairly well reproduced by decreasing the preactivation rate constant δ_2_ with a relatively small increase in the “unflipping” rate constant γ_2_ (Figure 5B). We have additionally observed that this modification of flipping/unflipping rates (δ_2_/γ_2_) predicted a slowdown of the current onset rate. Such a tendency to increased rise time was indeed observed experimentally, but it needs to be stressed that our recordings were performed in the lifted cell configuration for which the exchange time may be insufficient to estimate this parameter with a high fidelity (as, e.g., in excised patches). To achieve the optimal reproduction of our experimental observations, additionally, the desensitization rate constants (d_2_ and r_2_) had to be slightly modified (Figure 5B). We cannot exclude some changes of unbinding rate constant (k_off_), but even in this scenario, changes in preactivation rate constants would be still necessary. Therefore, we decided to limit our simulations to minimum requirement kinetic model postulating a major change in the flipping transition. Importantly, the major impact of the β_2_E155 mutation, related primarily to the preactivation process, was confirmed by simulations of superagonism of MSC and the upregulation of current responses to 300 mM GABA by FLU. Indeed, distinct MSC effect on gating in comparison to GABA (Figure 3) could be best reproduced for MSC by changing the flipping rate constants (δ_2_/γ_2_) toward those determined for the WT receptors (Figure 5B). Similarly, the following are the effects of FLU: the upregulation of current amplitude and fading, acceleration of current onset, and prolongation of deactivation kinetics for currents evoked by 300 mM GABA could be properly modeled by increasing the flipping rate δ_2_ with a small change in the desensitization rate d_2_ (Figure 5B). Altogether, a proper reproduction of the impact of the β_2_E155 mutation on the receptor gating required primarily a modification in the preactivation rate constants with a minor change in desensitization. Our simulations did not indicate any change in the opening/closing rates, and this conclusion is supported by our single-channel analysis and modeling (see below).

### Single-Channel Analysis Reveals Changes in Shut Time Distributions for Mutated Receptors Activated by GABA or MSC

Since the kinetic model used in the present study consists of several transitions (although it is still simplified) with a number of rate constants, which are typically difficult to be reliably optimized based solely on the macroscopic recordings, we extended our investigations by the single-channel analysis. We found that whereas in macroscopic recordings, short applications of 300 mM did not induce any visible deterioration of the signal stability, during much longer cell-attached single-channel recordings, at this concentration, instability of recordings was observed. We thus decided to carry out the single-channel recordings at 100 mM GABA concentration (EC_43_). Importantly, the single-channel activity induced by 100 mM GABA and mediated by the β_2_E155 mutants took clearly the form of clusters (Figure 6B). For the sake of comparison, the activity of WT receptors was monitored at a similar EC value, which was 30 μM GABA (EC_32_). Additionally, we performed experiments on β_2_E155 mutants using 8 mM MSC, which corresponds to an analogous EC value (EC_41_). Clearly, these minor differences in agonist potencies were taken into account in model simulations (see below). Figure 6 shows typical single-channel traces as well as exemplary open and shut time distributions for WT receptors (30 μM GABA) and the β_2_E155 mutant (100 mM GABA and 8 mM MSC). Open time distributions consisted of typically three components, and no significant differences in the distribution parameters were observed when comparing the activity of WT and mutated receptors (Table 1). Moreover, in the case of activation of the mutated receptors by MSC (8 mM), no difference was observed in the open times distributions in comparison to WT and 30 μM GABA and 100 mM GABA for mutated receptors (Table 1). In the case of shut time distributions, fitting of at least four components was needed. We have limited our statistics to the three shortest shut time components, i.e., the fourth one was omitted as it is likely to be affected by the presence of more than one channel within the patch (Kisiel et al., 2018). Taking into account nonsaturating conditions in our experiments, we may expect to observe bursting activity, and at least two shortest shut components are expected to reflect the activity of a fully bound receptor (Kisiel et al., 2018). For GABA-evoked activity, in the case of a mutant, we found a significant prolongation of the second shut time component as well as a decrease in its percentage compared to WT (Table 1). Interestingly, when MSC was applied to the mutated receptors (8 mM), the time constant of the second component in the shut time distributions decreased with respect to the value determined for GABA, showing thus a trend toward that observed for WT receptors (Table 1). Additionally, as expected from our macroscopic data, the β_2_E155 mutation significantly decreased P_open_ in bursts and clusters for GABA-evoked activity (Table 1). When applying MSC, P_open_ calculated for both bursts and clusters for mutated receptors was larger than in the case of GABA and became not significantly different from P_open_ determined for WT receptors activated by GABA (Table 1).

**Table 1:** Parameters of single-channel recordings.

Open times	P_1_	τ_1_ (ms)	P_2_	τ_2_ (ms)	P_3_	τ_3_ (ms)	τ_open_ (ms)
WT 30 μM GABA	0.20 ± 0.03	0.16 ± 0.03	0.53 ± 0.06	1.07 ± 0.17	0.27 ± 0.05	2.36 ± 0.35	1.25 ± 0.22
MUT 100 mM GABA	0.28 ± 0.05	0.17 ± 0.07	0.62 ± 0.03	0.82 ± 0.21	0.13 ± 0.06	2.67 ± 0.95	0.75 ± 0.17
MUT 8 mM MSC	0.28 ± 0.04	0.23 ± 0.03	0.58 ± 0.03	0.91 ± 0.10	0.33 ± 0.07	2.62 ± 0.25	1.19 ± 0.10
**Shut times**	P_1_	τ_1_ (ms)	P_2_	τ_2_ (ms)	P_3_	τ_3_ (ms)
WT 30 μM GABA	0.36 ± 0.03	0.045 ± 0.009	0.36 ± 0.02	0.21 ± 0.04	0.15 ± 0.02	1.25 ± 0.29
MUT 100 mM GABA	0.33 ± 0.07	0.063 ± 0.011	0.18 ± 0.01*	0.52 ± 0.10*	0.28 ± 0.07	3.97 ± 1.04
MUT 8 mM MSC	0.44 ± 0.05	0.062 ± 0.011	0.28 ± 0.04	**0.31 ± 0.04#**	0.22 ± 0.03	**1.46 ± 0.30#**
**Open probability**	**In clusters**			**In bursts**		
WT 30 μM GABA	0.472 ± 0.073			0.662 ± 0.040		
MUT 100 mM GABA	0.098 ± 0.011*			0.377 ± 0.087*		
MUT 8 mM MSC	**0.431 ± 0.010#**			0.548 ± 0.048			

Note: P, area of open and shut time distribution fitted with sum of exponentials; τ, shut and open time constants in these distributions, single-channel conductance, open probabilities estimated for clusters of bursts isolated manually and for bursts (t_crit._ between τ_3_ and τ_4_). Presented parameters were obtained for 300 mM GABA for wild-type (WT) α_1_β_2_γ_2_ receptors, 100 mM GABA for α_1_β_2_E155Cγ_2_ receptors and for 8 mM MSC for α_1_β_2_E155Cγ_2_ receptors. Data were calculated from 4 to 8 patches in each group as the mean ± SEM. *p* < 0.05 was marked by *—for comparisons between GABA-evoked currents in WT and mutants (MUT) or #—for comparisons between GABA- and MSC-evoked currents in mutants, and underlined by bold text. For reasons presented in Kisiel et al. (2018), the longest closures were not presented. p ≤ 0.05: * WT GABA vs. MUT GABA, # MUT GABA vs. MUT MSC.

Based on the NSVA, the β_2_E155 mutation did not affect the single-channel conductance of events elicited by GABA for WT and for mutants. Taking advantage of our single-channel recordings, we found that indeed the amplitude of single-channel currents recorded at the same holding voltage (100 mV) for WT receptors and for the mutants did not show any statistically significant difference (WT: A_GABA_ = −1.93 ± 0.17 pA, *n* = 12; MUT: A_GABA_ = −2.09 ± 0.08 pA, *n* = 20; *p* = 0.361, Table 1). Moreover, the amplitudes of single-channel currents elicited by MSC for mutated receptors were not statistically different from those determined for GABA-evoked currents (A_MSC_ = −2.24 ± 0.07 pA, *n* = 8 vs. MUT GABA *p* = 0.274). Thus, these single-channel data further confirm that neither the receptor mutation nor the use of different agonists (GABA, MSC) altered the single-channel conductance.

In our recent study (Kisiel et al., 2018), we found that alterations of the second shut time component are indicative for changes in the preactivation transition (δ_2_ and γ_2_). To further explore this prediction, we performed single-channel simulations. It needs to be emphasized that because of nonsaturation, both binding steps and the activity of singly bound receptors should be considered. We made an estimation that at 30 μM GABA, singly bound receptors are expected to contribute to approximately 13% of events characterized by short open time duration (data not shown). In our analysis, this percentage was certainly much lower as only clusters were selected for analysis. Indeed, in the open time distributions, the percentage of short-living openings is minor (Table 1). We thus simplified the model by omitting the singly bound states (Figure 7A), and only rate constants for doubly bound states were set free in the fitting procedure. For the WT receptors, the rate constants for the binding step (k_on_ and k_off_) were taken from Kisiel et al. (2018), and for the β_2_E155C mutant, we rescaled the k_on_ for the WT receptors by a factor resulting from the analysis of the dose–response relationships for WT receptors and the mutant. Thus, for macroscopic and single-channel simulations, the binding rate (k_on_) determined in model simulations for WT by Szczot et al. (2014) for macroscopic currents and by Kisiel et al. (2018) in the single-channel simulations, was reduced by ~2,000-fold. In Table 2 and Figure 7B, we show the results of such single-channel activity fitting for WT receptors and for the considered mutant. Notably, the only significant difference between rate constants (except for the binding step) determined for these receptors was a reduction in the flipping rate δ_2_ in the mutant, which is in agreement with our macroscopic analysis. Moreover, model fitting for activity mediated by the mutant and elicited by 8 mM MSC indicated a significant increase in the flipping rate δ_2_ with respect to that determined for GABA in the same receptor (Table 2, Figure 7B). This result further confirms that mutation of the β_2_E155 residue results in downregulation of the flipping rate, and the superagonism of MSC is due to upregulation of the preactivation rate δ_2_. However, it needs to be mentioned that besides a major change in δ_2_ in the case of mutant, there was also a relatively minor change in the α_2_′ rate constant (Table 2, Figure 7B).

**Table 2:** Kinetic model simulations for the single-channel activity based on single-channel kinetic model scheme shown in Figure 7A.

Rate constants	WT GABA	MUT GABA	MUT MSC
k_on_ [mM^−1^ ms^−1^]	43.2	0.0216	0.0432
k_off_ [ms^−1^]	1.82	1.82	1.82
*δ*_2_ [ms^−1^]	11.06 ± 1.22	2.18 ± 0.33*	**10.97 ± 3.04^#^**
*γ*_2_ [ms^−1^]	8.61 ± 1.01	5.81 ± 1.28	2.70 ± 0.76
*β*_2_ [ms^−1^]	8.48 ± 1.86	7.23 ± 3.14	7.61 ± 1.43
*α*_2_ [ms^−1^]	5.39 ± 1.70	4.78 ± 0.86	2.67 ± 0.37
*β*’_2_ [ms^−1^]	6.57 ± 0.61	3.91 ± 1.37	3.03 ± 0.66
*α*’_2_ [ms^−1^]	0.84 ± 0.11	1.12 ± 0.14	**0.59 ± 0.15^#^**
d_2_ [ms^−1^]	2.98 ± 0.25	4.15 ± 0.87	3.63 ± 0.43
r_2_ [ms^−1^]	4.00 ± 0.43	2.77 ± 0.46	3.26 ± 0.59
d’_2_ [ms^−1^]	0.77 ± 0.24	1.03 ± 0.36	1.49 ± 0.56
r’_2_ [ms^−1^]	0.03 ± 0.01	0.01 ± 0.01	0.47 ± 0.17

Note: the table presents the rate constant values for GABA-evoked currents for WT (column WT GABA) and for GABA or MSC-elicited currents for mutated receptors (MUT). Values in bold represent significant difference marked with *for WT GABA vs. MUT GABA and with # for MUT GABA vs. MUT MSC. p ≤ 0.05: *WT GABA vs. MUT GABA, # MUT GABA vs. MUT MSC.

## Discussion

The most important conclusion from the present study is that the β_2_E155C mutation causes not only a particularly strong effect on the receptor agonist binding but also a marked impact on the receptor gating. This is not surprising as some other mutations located within or in the close vicinity of the orthosteric binding site may strongly affect the receptor gating (e.g., Boileau et al., 2002; Newell et al., 2004; Laha and Wagner, 2011; Colquhoun and Lape, 2012; Laha and Tran, 2013; Szczot et al., 2014; Kisiel et al., 2018). Thus, these data further reinforce the view that structural elements of the receptor, which affect binding or gating are intermingled, and often, the same residues are involved in both aspects of the receptor activation.

When fitting the dose–response relationships, we were extracting the EC_50_ values, but surprisingly, the Hill coefficients (often referred to the number of the binding sites) is smaller than 1 (0.5–0.76, see legend for Figures 1, 3). This issue was thoroughly discussed in our previous article (Mozrzymas et al., 2003)—in principle, the Hill’s coefficient is informative about the number of binding sites when the scheme is limited to the binding reaction.

While some previous studies implicated the role of β_2_E155 residue in agonist binding (Newell et al., 2004; Mortensen et al., 2014), the impact of its mutation on gating is novel and of special interest. Our data provide a particularly solid evidence that the β_2_E155C mutation had a marked effect on the preactivation, which is strongly supported not only by a thorough analysis of macroscopic and single-channel traces evoked by GABA but also by the effect of FLU and by comparing currents evoked by GABA or MSC. Indeed, FLU caused an increase in amplitude, enhanced fading, accelerated onset, and prolonged current deactivation (Figures 4A–C), which can be regarded as hallmarks of accelerated preactivation rate (Szczot et al., 2014; Jatczak-Śliwa et al., 2018). In particular, these findings are in agreement with our recent study (Jatczak-Śliwa et al., 2018) where similar kinetic observations were made for a modulatory effect of FLU in the case of the α_1_F64 mutants that we could simulate by modifications of the preactivation and desensitization rate constants. Similarly, a robust increase in absolute amplitude of currents mediated by mutants and elicited by 30 mM MSC with respect to those evoked by 100 mM GABA (nearly equipotent doses for the mutated receptor, comparisons between GABA and MSC-evoked responses were made on the same patches; Figure 3) also indicated the upregulation of prectivation transition in our model simulations.

This coincidence that the mutation of β_2_E155 residue affects primarily binding and preactivation further supports the view that, as postulated by our models, preactivation is indeed the most likely transition, which follows the agonist binding and that β_2_E155 is an important part of a molecular pathway in which the energy supplied by the agonist binding is being conveyed to structures involved in preactivation transitions. On the other hand, as mentioned above, this is not a unique residue, which is important both in agonist binding and gating. Such a “dualistic” role of specific residues was described also for the amino acids belonging to the part of the binding site located at the complementary subunit (e.g., Newell and Czajkowski, 2003; Szczot et al., 2014). Thus, the present findings reinforce the emerging picture that from a physically large structure of the agonist-binding site, the mechanical signal leading to the pore opening is conveyed by several molecular pathways within both principal and complementary subunits.

Notably, our data related to the weakening of the agonist-binding site caused by the β_2_E155C mutation are in qualitative agreement with results of other groups, although there were also some differences among different receptors providing additional insight into the role of this residue in binding and gating properties. Newell et al. (2004) performed experiments on α_1_β_2_E155C receptors (αβ receptor type) expressed in oocytes and observed a 3,375-fold lower potency of GABA. However, it needs to be stressed that we studied αβγ-type receptors. These receptor types are known to show different properties including affinity, kinetics, conductance, and susceptibility to pharmacological modulation (Horenstein and Akabas, 1998; Scheller and Forman, 2001; Wagner et al., 2004; Wilkins et al., 2005; Mercik et al., 2006). In addition, Mortensen et al. (2014) showed around 400-fold increase in EC_50_ value (relative to WT) for a different substitution of the E155 residue for receptors (α_1_β_2_E155Qγ_2S_) expressed in HEK cells. Our findings (of a 1,836-fold increase in EC_50_) are thus in qualitative agreement with studies by Newell et al. (2004) and Mortensen et al. (2014). This agreement indicates that the impact of the β_2_E155 mutation is qualitatively similar for αβγ and αβ receptor types, suggesting that impairment of the receptor affinity is mostly related to alterations occurring locally within or close to the orthosteric agonist-binding sites.

Recordings of the spontaneous macroscopic activity using picrotoxin (Figures 2C1,C2) revealed that the β_2_E155C mutant generates much larger spontaneous currents than the WT receptors providing further evidence that this mutation indeed affects the receptor gating. A similar observation was reported by Newell et al. (2004) and Mortensen et al. (2014). It is known that β subunits can form the spontaneously active (i.e., without GABA) homomers (Blair et al., 1988; Cestari et al., 1996; Wooltorton et al., 1997), which could contribute to the observed spontaneous activity. However, it needs to be stressed that functional homomers were demonstrated for the β_3_ (Connolly et al., 1996; Davies et al., 1997; Wooltorton et al., 1997; Gottschald Chiodi et al., 2019) and β_1_ (Sigel et al., 1989; Sanna et al., 1995; Krishek et al., 1996; Miko et al., 2004) subunits, whereas in the present study, β_2_ subunits were expressed. Moreover, the expression level of putative β subunit homomers is expected to be the same in the case of WT and the mutants, and the spontaneous activity in the case of the WT receptors was negligible indicating that contribution from the β homomers is low if any.

It is known that in the case of WT receptors, macroscopic currents elicited by saturating concentrations of GABA or MSC differ only in deactivation kinetics (Jones et al., 1998; Szczot et al., 2014). These data predict that for currents mediated by the WT and elicited by EC_74_ of GABA or by EC_77_ of MSC would show an approximately twofold increase (in the case of MSC) in the slow deactivation time constant, which is similar to what we have found at saturating concentrations of these agonists in the case of WT receptors (Szczot et al., 2014). This observation indicates that within these concentration ranges, key kinetic parameters are likely to be similar to what is observed at saturation. In our study on the α_1_β_2_E155Cγ_2_ mutant, however, no difference in the deactivation kinetics for GABA and MSC was observed. Moreover, deactivation process in the case of mutants was nearly one order of magnitude faster than in the case of WT receptors. This observation suggests that the β_2_E155C mutation so strongly altered binding and gating features underlying the deactivation process, that in mutants, differences in deactivation for GABA and MSC were not observed in sharp contrast to the WT receptors. In addition, our simulations of MSC-elicited currents indicate that this agonist acts as a superagonist because of upregulation of the preactivation transition (see also, e.g., Figure 3B and Szczot et al., 2014). Thus, as also mentioned above, our analysis based on both macroscopic and microscopic currents provides solid evidence that observed kinetic alterations caused by the β_2_E155C mutation result from changes in preactivation kinetics rather than from a “side-effect” of nonsaturating conditions. Interestingly, while our simulations indicate that β_2_E155C mutation primarily downregulated the preactivation rate (δ_2_ with a minor change also in γ_2_), in our previous study (Szczot et al., 2014), we found that in the case of WT or α_1_F64 mutants, the largest effect concerned the γ_2_ (unflipping) rate constant. This observation may indicate the fact that mutation of different residues (at different subunits) might influence preactivation process in a different way.

Our data show that the considered β_2_E155C mutation dramatically affects binding with a strong effect on gating (preactivation), but it does not affect the receptor single-channel conductance. The last conclusion is supported by two lines of evidence—NSVA and single-channel recordings. Notably, this is not an uncommon situation as the majority of considered mutations affected primarily the binding and gating properties of GABA_A_R with a minor if any effect on conductance (see, for example, Laha and Wagner, 2011; Szczot et al., 2014).

These data are an important step forward in deciphering the molecular mechanisms of GABA_A_R activation. It needs to be emphasized that this research, by adding to the field of investigations into the relation between structure and function of the GABA_A_R, are likely to be useful in designing drugs exerting clinically expected effects. As we have already mentioned, the β_2_E155 residue is important in pathophysiological context as its mutation is linked with childhood absence epilepsy (Epi4K and EPGP Investigators, 2013). Moreover, a special role of the β_2_E155 residue, mainly in binding and preactivation transition, reported in this study indicates that some amino acids located at strategic positions within the GABA_A_R structure might show strong specialization in regulation of specific conformational transitions. However, as already mentioned, it needs to be stressed that in spite of emerging (mainly) static structures of GABA_A_R (Miller and Aricescu, 2014; Phulera et al., 2018; Zhu et al., 2018), the molecular mechanisms of GABA_A_R activation remain elusive. Further studies on the role of specific amino acid residues in the GABA_A_R activation are needed, and it may be expected that when this body of evidence reaches a “critical mass, the activation mechanism will be finally revealed. In conclusion, we provide the first evidence that the β_2_E155C mutation strongly affects the GABA_A_R gating, having the largest impact on the preactivation transition.

## Conclusions

Macroscopic and single-channel analyses were used to address the impact of the β_2_E155 mutation on the channel binding and gating. Marked rightward shift of the dose–response and alterations in the shut time distribution indicated a strong impact on the binding process combined with alteration of gating, especially the preactivation transition. Involvement of the β_2_E155 residue in preactivation transition was further supported by the analysis of the mutant’s modulation by flurazepam (FLU). In addition, muscimol (MSC) was found to act as a superagonist for the β_2_E155 mutant by reinforcing preactivation with respect to GABA, further underscoring that this transition is affected by the β_2_E155 mutation. Altogether, we provided novel evidence that the β_2_E155 residue is involved not only in agonist-binding process but also in the receptor gating transitions, primarily preactivation.

## Data Availability Statement

Datasets are available on request. Please contact corresponding authors.

## Author Contributions

MJ-Ś participated in the designing and performing the experiments, data analysis, all model simulations, and writing the article. MK participated in the designing and performing the experiments, data analysis, and writing the article. MC participated in performing some experiments, data analysis, and wrote a part of the article. MB participated in performing some experiments and data analysis. JM conceived the project, designed the research, supervised the experiments, data analysis, and model simulations, wrote the article, and edited the final version of the manuscript, procured the main body of funding.

## Conflict of Interest

The authors declare that the research was conducted in the absence of any commercial or financial relationships that could be construed as a potential conflict of interest.

## Abbreviations

GABA_A_R: ionotropic GABAergic receptor, type A
WT: wild-type of α_1_β_2_γ_2_ GABA_A_ receptor
MUT: β_2_E155C mutant of α_1_β_2_γ_2_ GABA_A_ receptor
MSC: muscimol
FLU: flurazepam, a benzodiazepine derivative
PTX: picrotoxin, open GABA_A_ receptor channel blocker
RT: rise time of currents mediated by GABA_A_ receptors upon activation
FR: fraction of the current that remained after selected time after the peak.
